# A two-dimensional ON/OFF switching device based on anisotropic interactions of atomic quantum dots on Si(100):H

**DOI:** 10.1038/s41467-017-02377-4

**Published:** 2017-12-20

**Authors:** Mayssa Yengui, Eric Duverger, Philippe Sonnet, Damien Riedel

**Affiliations:** 1 Institut des Sciences Moléculaires d’Orsay (ISMO), CNRS, Univ. Paris Sud, Université Paris-Saclay, 91405 Orsay, France; 2 Institut FEMTO-ST, Univ. Bourgogne Franche-Comté, CNRS, 15B avenue des Montboucons, 25030 Besançon, France; 3 Institut de Science des Matériaux de Mulhouse (IS2M), CNRS, UMR 7361, Université de Haute Alsace, 3 bis rue Alfred Werner, 68057 Mulhouse, France

## Abstract

Controlling the properties of quantum dots at the atomic scale, such as dangling bonds, is a general motivation as they allow studying various nanoscale processes including atomic switches, charge storage, or low binding energy state interactions. Adjusting the coupling of individual silicon dangling bonds to form a 2D device having a defined function remains a challenge. Here, we exploit the anisotropic interactions between silicon dangling bonds on n-type doped Si(100):H surface to tune their hybridization. This process arises from interactions between the subsurface silicon network and dangling bonds inducing a combination of Jahn–Teller distortions and local charge ordering. A three-pointed star-shaped device prototype is designed. By changing the charge state of this device, its electronic properties are shown to switch reversibly from an ON to an OFF state via local change of its central gap. Our results provide a playground for the study of quantum information at the nanoscale.

## Abstract (web summary)

Using scanning tunnelling microscopes to manipulate atomic quantum dots at surfaces enables the creation of devices using inter-dot interactions. Here the authors demonstrate the design and operation of nanoscale ON/OFF switches by arranging four silicon dangling bonds.

## Introduction

The use of scanning tunneling microscopy (STM) has played a decisive role in the race to control atomic-scale quantum dots (QDs) at surfaces^1–5^ during the last few decades. In this context, the study of the electronic properties of dangling bonds as QD on the Si(100):H^6–10^, Ge(100):H^11^, or doped Si(111):B^12^ surfaces appears to be a powerful alternative to other larger systems due to their suitable electronic states whose energies are located in the surface band gap^13,14^. Remarkable progress has highlighted several of their physical or chemical characteristics^15–18^ demonstrating the intimate and subtle interactions between the semiconductor bulk and the substrate surface properties^19,20^ including subsurface dopants^9,16^. The fundamental structure of semiconductor surfaces can lead to particular delocalized or localized charge density distributions^21,22^ and reconstruction phases at low temperature^23,24^. Similar properties have also been reported when localized at silicon dangling bonds (Si-DB(s))^25^. The easily controlled fabrication of Si-DB QDs with STM has attracted intense investigations^26,27^ such as the exploration of organized spatial arrangements similar to cellular automata at room temperature (300 K)^17,28^. In these structures, long range Coulomb interactions are exploited to control the variations of the electronic charge state at each Si-DB in the cell^29^. More recently, other experiments performed at lower temperature (77 K) on the n-type doped Si(100):H surface show that the electronic interactions between Si-DBs periodically spaced by a hydrogenated silicon dimer lead to the formation of excited empty states as low binding energy states^18^. This aforementioned work reported the observation of an unoccupied charge density located in between the Si-DBs. Beyond the observed differences between these two previous works performed at room and low temperatures, the general use of the Si-DB QD interactions as devices^30,31^ requires to take into account the correlation between the electronic properties of the surface and the ones of the Si-DB states^4^, and in particular, the role of the subsurface dopants^32^.

Here we present an STM investigation of coupled Si-DB structures showing that the anisotropic electronic interactions between the QDs at 9 K are intrinsically related to the silicon surface properties. A precise comparison between our experimental data and numerical simulations that use the density-functional theory (DFT) demonstrates that the initial charge transfer between the As dopant atoms and the Si-DBs induce local Jahn–Teller distortions of the silicon lattice within the Si-DBs. Consequently, a charge density located in between the Si-DBs is formed via a charge ordering effect that allows the QDs to hybridize. The coupling charge density is shown to be different when the Si-DBs are aligned along the [$11$0] and [$1\bar 1$0] crystallographic directions of the Si(100):H surface. By using these properties in a device made of four Si-DBs, we can optimize the hybridization between the Si-DBs to control a reversible ON/OFF switching function via opening a local gap at the central part of the device.

## Results

### Anisotropic aspects of the Si-DB QD interactions

**Fig. 1:**
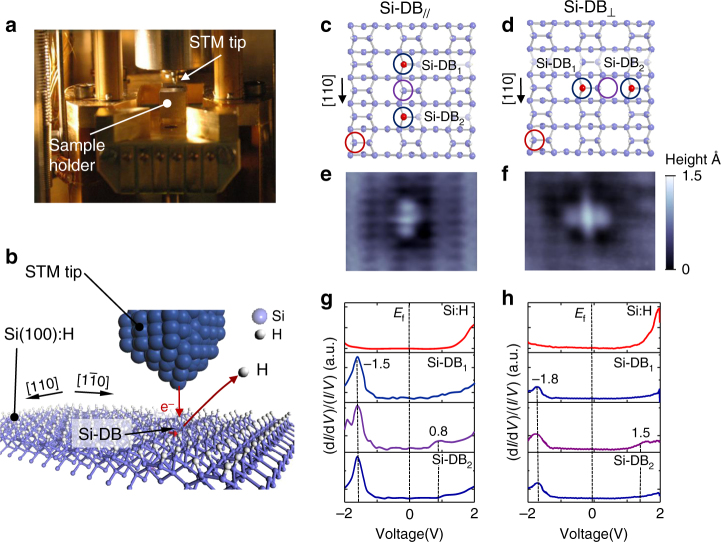
Topographical and electronic aspects of Si-DB interactions. **a** Photography of the inside scanner of the low-temperature (9 K) scanning tunneling microscope. **b** ball and stick sketch of the Si(100):H surface excited by the tungsten tip of the microscope during the local desorption process of an H atom. **c**, **d** Ball and stick sketches of the Si-DB_//_ and Si-DB_⊥_ made of two Si-DBs oriented parallel (**c**) or perpendicular (**d**) to the [110] direction of the silicon lattice. The blue, purple, and red circles indicate the positions where the (d*I*/d*V*)/(*I*/*V*) measurements are done. **e**, **f** (34.6 × 36 Å^2^) STM topographies (*V* = +1.7 V, *I* = 33 pA) of the corresponding Si-DB_//-⊥_ having two Si-DBs separated by a fully hydrogenated silicon dimer. **g**, **h** Normalized (d*I*/d*V*)/(*I*/*V*) curves measured on the Si-DB_//_ and Si-DB_⊥_. The (d*I*/d*V*)/(*I*/*V*) curves on the Si:H, the Si-DB positions, and in between the Si-DB are red, blue, and purple, respectively

For this work, we have used a Beetle-Besocke STM running at 9 K (Fig. 1a). The high spatial resolution and stability of this tool provides sufficient spatial precision to desorb at will hydrogen atoms from the Si(100):H surface in order to fabricate any requested spatial distribution of Si-DBs on the surface (Fig. 1b). From a topographical point of view, the empty-state STM image of two coupled Si-DBs oriented along or perpendicular to the silicon dimer rows, when separated by a fully hydrogenated Si dimer (Fig. 1c, d), show clear differences of spatial charge density distribution (Fig. 1e, f). The bright protrusion located in between the Si-DBs, which is one apparent signature of the Si-DB coupling at low temperature^18^ is more elongated in the perpendicular case (Fig. 1f) than in the parallel case (Fig. 1e). In the following, we will write Si-DB_//_ or Si-DB_⊥_ to label the Si-DB lines that are oriented along or perpendicular to the silicon dimers rows, respectively. The expression Si-DB_//-⊥_ designates the case where both directions are considered.

The anisotropic interaction between Si-DBs can be also pointed out via the measurement of the (d*I*/d*V*)/(*I*/*V*) signal along the spatial distribution of charge density at the Si-DB_//-⊥_. In the case of the Si-DB_//_ (Fig. 1g), the (d*I*/d*V*)/(*I*/*V*) curves show a major peak of density of state (DOS) at ~−1.5 V in the valence band and a single peak of DOS at ~0.8 V in the conduction band. The (d*I*/d*V*)/(*I*/*V*) spectra of the Si-DB_⊥_ are significantly different (Fig. 1h). The DOS peaks observed in the valence band are shifted to lower energy (i.e., −1.8 V) along the Si-DB_⊥_, whereas the conduction band exhibits a DOS peak at 1.5 V in between the Si-DBs.

**Fig. 2:**
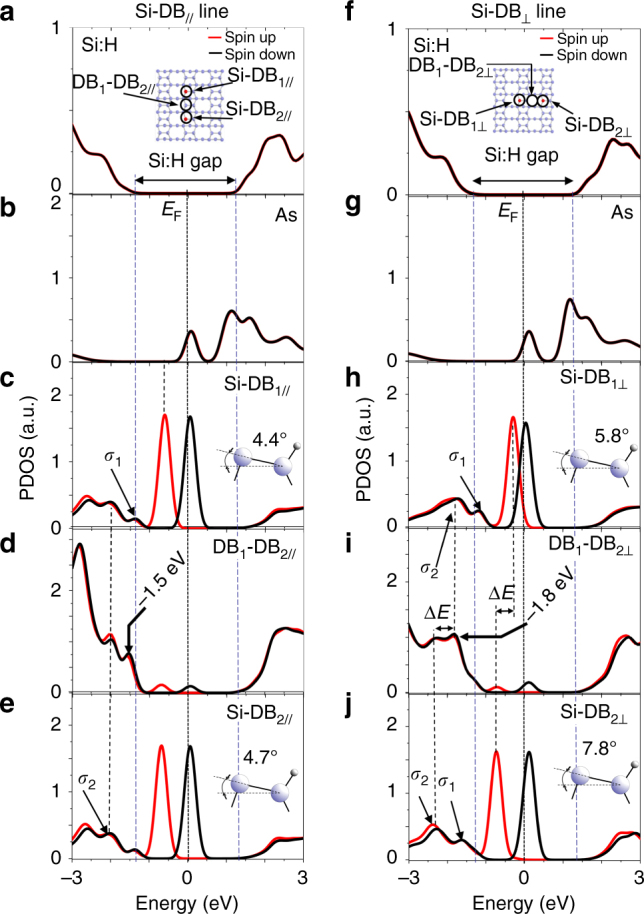
Numerical analysis of the Si-DB electronic structure. **a**–**e** PDOS curve series calculated on the Si–H bond, the As atom, the Si-DB_1_, the Si–H bond in between the Si-DBs and the Si-DB_2_ positions of the Si-DB_//_, respectively. **f**–**j** PDOS curve series calculated with the same sequence as in **a** for the Si-DB_⊥_. The positions of the involved atoms are indicated in the inset of **a** and **f**. The blue and white balls of the silicon slab insets are Si and H atoms, respectively. The Si atoms having a Si-DB are represented in red. Note that the position of the As atom is not indicated in the inset in **a** or **f** as it is located far from the Si-DB (see Supplementary Fig. 11 for more details). The red and black curves describe the spin-up and spin-down density of state, respectively

Density-functional theory simulations performed on the Si(100):H surface for two Si-DB_//-⊥_ oriented in the [$11$0] and [$1\bar 1$0] directions provide spin-polarized partial density of state (PDOS) curves (Fig. 2). At the As dopant atom (not shown in the insets of Fig. 2a, f), the two curves are similar for both Si-DB_//-⊥_ directions and show four main PDOS peaks: one centered at *E*_F_ and three other peaks spreading over the silicon conduction band (Fig. 2b, g). The PDOS curves located at the two Si-DBs of the Si-DB_//_ are similar and show a spin-up PDOS peak below *E*_F_ and a spin-down PDOS peak pinning the Fermi level with the As state (Fig. 2c, e). In the valence band, the PDOS curves exhibit two valence *σ*_1_ and *σ*_2_ bands that can be related to subsurface Si–Si back-bond bands of the pristine Si(100) surface. Interestingly, at the middle of the Si-DB_//_ (Fig. 2d), the PDOS peaks of the Si-DB states at *E*_F_ can still be observed in the silicon band gap and the valence band PDOS peak *σ*_1_ is slightly shifted to lower energies (−1.5 V) and clearly corresponds to the (d*I*/d*V*)/(*I*/*V*) peak measured in Fig. 1g.

The PDOS curves considered at the Si-DB_⊥_ (Fig. 2f–j) do not show the same type of similarities. Firstly, there is a lift of degeneracy between the spin-up PDOS peaks of the Si-DB_1⊥_ and Si-DB_2⊥_, whereas the spin-down PDOS peaks remain within the Si(100):H Fermi level energy (*E*_F_). The degeneracy lift energy Δ*E* of the two spin-up states can be correlated with the energy shift of the *σ*_2_ valence bands at the Si-DB_1⊥_ and Si-DB_2⊥_ indicating that the electronic structure of the interacting Si-DBs significantly modifies locally the valence band of the Si(100):H surface. As a result, the signature of the electronic coupling at the center of the Si-DB_⊥_ exhibits a PDOS peak centered at −1.8 V, reproducing our experimental findings in Fig. 1h. A summary of the Si(100) surface band energy values is provided in the Supplementary Table 1.

These data can be linked with the tilt angle of the silicon dimer holding the Si-DB (see insets in Fig. 2c, e for Si-DB_//_ and 2h, j for Si-DB_⊥_). A careful analysis of these angles shows comparable values for the Si-DB_//_ (*α*_Si-DB1//_ = 4.4° and *α*_Si-DB2//_ = 4.7°), whereas the angles are significantly different in the case of the Si-DB_⊥_ (*α*_Si-DB1⊥_ = 5.8° and *α*_Si-DB2⊥_ = 7.8°). Previous works indicate that for an n-type-doped Si(100):H sample, the silicon dimers holding a negatively charged Si-DB is tilted so that the Si radical points upward. The same silicon dimer would point downward when neutral (for p-type-doped samples)^16^. In the present case, each of the Si-DBs considered in the Si-DB_//-⊥_ are shown to be negatively charged in the same way because of the presence of the As atom in the silicon slab^9^. This indicates that the charge transfer between the As atom and the Si-DBs is not at the origin of the degeneracy lift observed at the Si-DB_⊥_. This process arises from the electronic anisotropic interaction between Si-DBs induced by the intrinsic structure of the reconstructed Si(100):H surface.

**Fig. 3:**
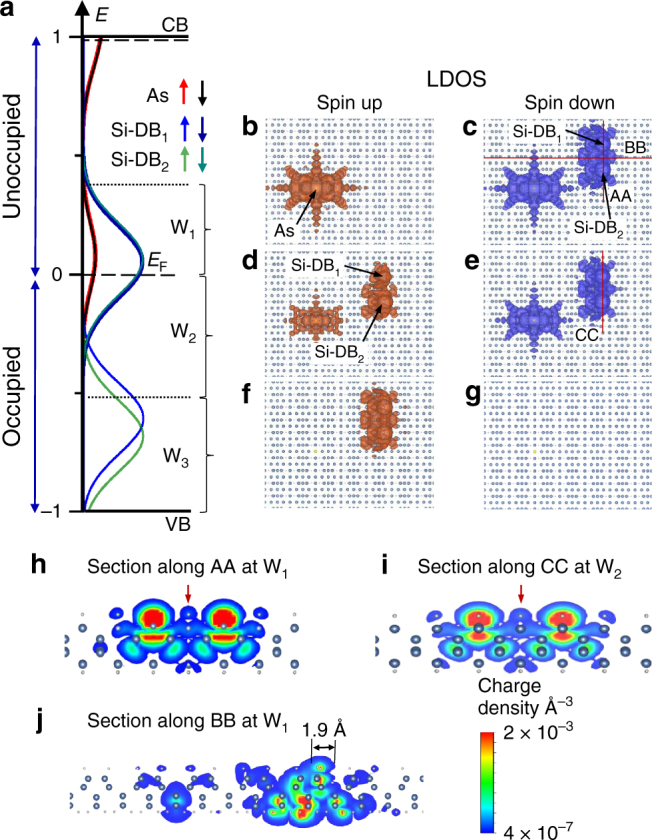
Calculated spatial distributions of charge for a Si-DB_//_. **a** PDOS curves at the two Si-DB_//_ and the As atom (recall from Fig. 2) for three selected energy windows W_1_–W_3_. **b**–**g** Spin-up (brown) and spin-down (blue) local density of state (LDOS) isosurfaces on the silicon slab for the three energy windows W_1_–W_3_. The chosen section of the slab is represented with blue and white balls as Si and H atoms, respectively. **h**–**j** Cross-sectional plot of the partial LDOS along the AA, BB, and CC axes defined by the red lines in **c** and **e**

**Fig. 4:**
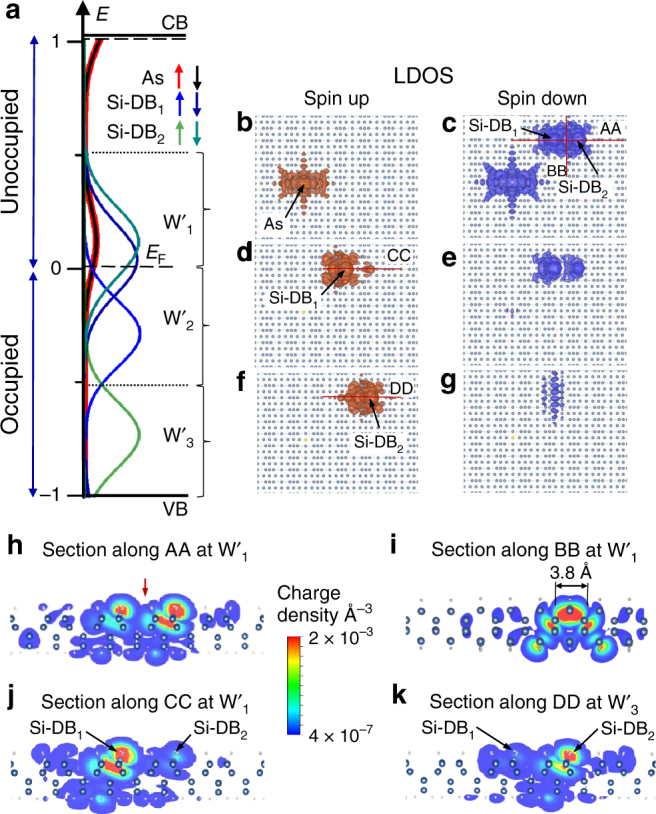
Calculated spatial distributions of charge for a Si-DB_⊥_. **a** Calculated PDOS curves at the two Si-DB_⊥_ and the As atom (recall from Fig. 2) for three selected energy windows W′_1_–W′_3_. **b**–**g** Spin-up (brown) and spin-down (blue) local density of state (LDOS) isosurfaces on the silicon slab for the three energy windows W′_1_–W′_3_. The chosen section of the slab is represented with blue and white balls as Si and H atoms, respectively. **h**–**k** Cross-sectional plot of the partial LDOS along the AA, BB, CC, and DD axes defined by the red lines in **c**, **d**, and **f**. The differences of hybridization of the Si-DBs between Figs. 3 and 4 can be clearly observed in Fig. 3d, f compared to Fig. 4d, f

The local density of state (LDOS) that reveals the spatial distribution of the calculated PDOS peaks are computed for three energy windows at the Si-DB_//_ (W_1_–W_3_) and Si-DB_⊥_ (W′_1_–W′_3_), and reported in Figs. 3 and 4. At W_1_ (Fig. 3a), the As atom is solely hybridized with the spin-down Si-DB empty state (Fig. 3b, c). The occupied part (W_2_) of the same PDOS peak exhibits similar spatial LDOS distributions (Fig. 3d, e) and traduces how the spin-down state of the Si-DB is incompletely occupied via a charge transfer from the As atom. The LDOS at W_3_ is thus simply related to the two quasi degenerated spin-up states located at the Si-DBs (Fig. 3f, g). We can underline the good matching between the experimental unoccupied STM topographies and the computed LDOS by looking at the sectional plots along the AA, BB, and CC axes that cross the Si-DB_//_ along two perpendicular directions (red lines in Fig. 3c, e). Figures 3h–j show the ensuing LDOS mapping in which one can clearly see a LDOS protrusion in the middle of the Si-DB_//_ (red arrows in Fig. 3h, i), as observed experimentally in Fig. 1e.

Similarly to what is observed with the Si-DB_//_, the PDOS peaks at W′_1_ (Fig. 4a) on a Si-DB_⊥_ show that the empty state of the As atom is pinned by the empty states of the Si-DB at *E*_F_ (Fig. 4b, c). At W′_2_ (Fig. 4e), the occupied part of the Si-DB spin-down LDOS is slightly asymmetric revealing the small degeneracy lift of the empty PDOS peaks of the Si-DB_⊥_ states. The STM topographies in Fig. 1f can also be reproduced by the cross-sectional LDOS maps along the two perpendicular AA and BB axes (red lines in Fig. 4c) that exhibit a LDOS protrusion located in the middle of the two dangling bonds (Fig. 4h, i). Now, if one compare the LDOS at W′_2_ and W′_3_ that include the two non-degenerated spin-up PDOS peaks of the Si-DB_⊥_, Fig. 4d, f show a clear spatial separation of the LDOS: one part of the spin-up LDOS at W′_2_ is mainly localized on the left side of the Si-DB_⊥_ (Si-DB_1_ in Fig. 4d) and the other spin-up LDOS part on the right side of the Si-DB_⊥_ at W′_3_ (Si-DB_2_ in Fig. 4f). The sectional drawings of the LDOS crossing the Si-DB_⊥_ along the CC and DD sections at W′_2_ and W′_3_ confirm this effect and reveal tilted LDOS intensities at each Si-DBs of the line (Fig. 4j, k) that extend to the neighbor Si-DBs. This is showing a clear signature of the Si-DB hybridization through the silicon subsurface.

A comparison of the calculated cross-sectional LDOS along two axes oriented perpendicular to the Si-DB_//_ and Si-DB_⊥_ show that the LDOS at the central protrusion spreads over ~3.8 Å in between the Si-DB_⊥_ (Fig. 4i) while it is confined within 1.9 Å on the Si-DB_//_ (Fig. 3j). These data further reproduce the trend of our experimental findings and show that the bright protrusion observed in the STM topographies intrinsically arise from the anisotropic interactions of the Si-DB QDs with the silicon surface states.

As described in unseparated Si-DB chains^33^, the coupling between two Si-DBs split by a Si–H dimer exhibits electronic and structural perturbations similar to Jahn–Teller distortion due to their negative partial charge state. Differently to unseparated Si-DB chains, the local alteration of the silicon lattice, in relation with the tilt of the silicon dimers holding the Si-DBs is here correlated with a charge ordering effect, revealing the observed Si-DB coupling protrusion in the unoccupied STM images. The charge ordering is hence different at the Si-DB_//_ and Si-DB_⊥_ due to the anisotropic reorganization of the Si-DB states and the locally perturbed Si(100):H valence bands. Therefore, our results indicate that the Si-DBs of the Si-DB_⊥_ are less efficiently hybridized than the Si-DB_//_. A more detailed analysis of these effects is given in the Supplementary Figs. 1–7 and Supplementary Notes 1–7.

It is striking to observe that the Si-DB PDOS peaks computed at the Fermi level energy of the Si(100):H surface are not detected experimentally^9,15,16^. Indeed, in the band gap of the silicon surface, there is no conducting channel that allows to probe these states at zero bias. However, we also observe that the unoccupied DOS peaks shown in the (d*I*/d*V*)/(*I*/*V*) curves in Fig. 1 are not directly reproduced in the simulated PDOS data computed in between the Si-DBs (Fig. 2d, i), whereas the LDOS of the Si-DB_//-⊥_ (Figs. 3c and 4c) reproduces accurately the shape of the experimental unoccupied-state STM topographies at *E*_F_. This difference can be explained when one considers the real aspect of the (d*I*/d*V*)/(*I*/*V*) signal measured at positive bias (i.e., in the conduction band) along the Si-DB_//-⊥_ as the result of an electronic transport of tunnel electrons flowing from the STM tip through the Si-DB states and the surface. The ensuing conducting channels are related to the unoccupied density of state at the silicon surface and subsurface, and in particular with the ones related to the As atom. As the positive bias gradually growths during the measurement of the (d*I*/d*V*)/(*I*/*V*) signal, the tip-induced band bending progressively increases at the Si(100):H surface^13,16,34^ inducing the Si-DB states, initially located at *E*_F_, to shift at higher energies, similarly to what is observed with a STM double junction barrier^18,35^. During this process, the DOS peaks measured experimentally in the conduction band at the coupling protrusions of the Si-DB_//-⊥_ (i.e., at 0.8 V and 1.5 V for the Si-DB_//_ and Si-DB_⊥_, respectively) can be related to the calculated unoccupied PDOS peaks at the As atom (i.e., ~1.1 eV and ~1.6 eV). Hence, the unoccupied DOS peaks observed in the (d*I*/d*V*)/(*I*/*V*) curves in Fig. 1 mostly arise from tunneling processes involving transport channels opening when the unoccupied Si-DB states energy match the ones of the As atoms, as it can be observed with gap states^36^. This effect is further demonstrated by the fact that no energy shift of the unoccupied Si-DB states is observed in the (d*I*/d*V*)/(*I*/*V*) curves at the coupling areas when the STM tip height decreases (Supplementary Fig. 8). As a result, the hybridized Si-DB states described at *E*_F_ via DFT simulations can be probed at higher positive biases with the STM. The unoccupied-state STM topographies of the Si-DB_//-⊥_ appear hence to be the result of two major processes: The first process arises from electronic transport through the spin-down empty state of the hybridized Si-DBs as described in Figs. 3c and 4c via the delocalized As states in the subsurface. The second effect is due to a contribution of the lateral electronic coupling through the delocalized spin-down empty states.

### A two-dimensional device using the anisotropic Si-DB interactions

**Fig. 5:**
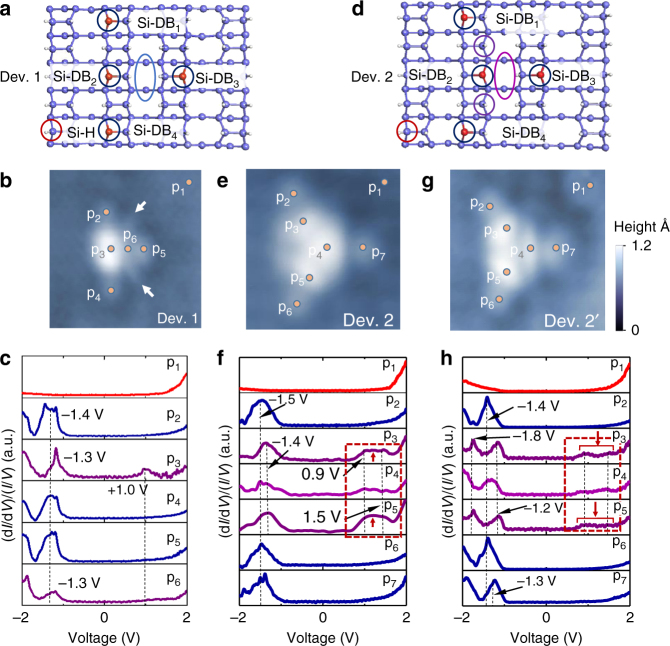
Electronic structure of the Si-DB devices Dev 1 and Dev 2. **a** Ball and stick sketch of the four Si-DB structures named Dev. 1. The blue and white balls are Si and H atoms, respectively. The Si atoms without H atom are represented in red. **b** (32 × 20 Å^2^) Unoccupied STM topography (1.8 V, 15 pA) of Dev. 1. **c** Normalized (d*I*/d*V*)/(*I*/*V*) curve series acquired on the points p_1_– p_6_ as described in **b**. **d** Ball and stick sketch of the four Si-DB structure named Dev. 2 with the Si and H atoms colored as in **a**. **e** (20 × 20 Å^2^) Unoccupied STM topography (1.8 V, 15 pA) of Dev. 2 before switching. **f** Normalized (d*I*/d*V*)/(*I*/*V*) curve series acquired on the seven positions p_1_–p_7_ as indicated in **e**. **g** (20 × 20 Å^2^) Unoccupied STM topography (1.8 V, 15 pA) of Dev. 2′ obtained after the excitation of Dev. 2. **h** Normalized (d*I*/d*V*)/(*I*/*V*) curves series acquired at the seven positions p_1_–p_7_ as indicated in **g**

It is now stimulating to explore how the anisotropic properties of interacting Si-DBs can be exploited to create a functionalized arrangement of QD in a two-dimensional (2D) structure. Here we propose to study the anisotropic effects in two devices made of four Si-DBs in a “T” or “Y” shape configurations. For the T shape, three Si-DBs are periodically located on the same silicon dimer row and the fourth one beside them (Fig. 5a). The ensuing empty-state STM topography of this device (Dev. 1) is shown in Fig. 5b and reveals a bright distribution of charge density centered in the middle of the Si-DB_//_ (point p_3_ in Fig. 5b) with a weaker curved charge density distributed in between the Si-DB_//_ and the fourth perpendicular Si-DB (see white arrows in Fig. 5b). The (d*I*/d*V*)/(*I*/*V*) measurements performed on this structure at various locations on the device (Fig. 5c) show a band of occupied DOS centered at ~−1.4 V for each measurement points except at the Si(100):H surface. Additionally, the single unoccupied DOS peak measured at point p_3_ is a signature of a dominant coupling of the Si-DBs along the Si-DB_//_ section of the device (Fig. 1g). Along the Si-DB_⊥_ section (Si-DB_2_ - Si-DB_3_), the coupling remains weak in Dev. 1 due to the larger distance between the Si-DB_2_ and Si-DB_3_ compared to the other Si-DBs of the Si-DB_//_. Coherently, the (d*I*/d*V*)/(*I*/*V*) signal at point p_6_ does not exhibit peak in the conduction band. To enhance the anisotropic Si-DB coupling in the [$1\bar 10$] direction, the Si-DB_2_ located in the middle of the Si-DB_//_ is moved to the right side of the Si dimer row, leading to the formation of a device with a “Y” shape named Dev. 2 (Fig. 5d). The change in the Si-DB position can be precisely done by applying a negative voltage pulse beside the concerned Si radical^15^. The corresponding empty-state STM topography of Dev. 2 shows a completely different spatial distribution of charge density having a three-pointed star shape (Fig. 5e). This device exhibits specific electronic properties as it depicts now an intense charge density protrusion in between Si-DB_2_ and Si-DB_3_, at the center of the structure (point p_4_ in Fig. 5e). As explained previously, the shape of the unoccupied-state STM image that partly arises from a lateral electronic coupling delocalized all along the Si-DB_//_ in Dev. 1 is now separated into two symmetric branches whose charge density protrusion are located at points p_3_ and p_5_ (Fig. 5e). In these conditions, the three Si-DBs forming a zig-zag shape in Dev. 2 is composed of two coupled pairs of Si-DB_//_ (Supplementary Fig. 7) that can hybridize to the Si-DB_⊥_ section of the device. The (d*I*/d*V*)/(*I*/*V*) spectroscopy curves acquired on Dev. 2 (Fig. 5f) confirm this electronic structure as they show additional intense unoccupied states at the points p_3_, p_4_, and p_5_ spreading from 0.9 to 1.5 V. It is the signature of an enhanced lateral electronic coupling arising from a combination of parallel and perpendicular interactions between the Si-DBs in Dev. 2. The slight shift of the occupied DOS peaks from −1.4 to −1.5 V at points p_2_, p_6_, and p_7_ also confirm the mixed interactions.

By applying voltage pulses (i.e., ~2.0 V) in the middle of Dev. 2 (point p_4_ in Fig. 5e), the STM topography of Dev. 2 changes to exhibit a new distribution of charge density as shown in the STM topography in Fig. 5g. The electronic state of the device is now called Dev. 2′. It is important to emphasize that the relative lateral positions of the Si-DBs have not changed between Dev. 2 and Dev. 2′ differently to the modifications between Dev. 1 and Dev. 2. In Dev. 2′, only the electronic structure changes compared to Dev. 2 (Supplementary Fig. 9). In particular, the distribution of the charge density is shrunk and elongated at point p_4_ in Dev. 2′ (Fig. 5g). The reversible Dev. 2′→Dev. 2 switch is obtained by scanning over the structure at negative bias with the STM tip (i.e., below −2.3 V) similarly to what can be done with a single Si-DB on p-doped substrates^16^. During the reversible switching processes Dev. 2↔Dev. 2′, although the tip-induced electrostatic field can play the role in shifting the energy levels of the Si-DB states, it is not considered as the main active process as it has no particular local effect on the device. The (d*I*/d*V*)/(*I*/*V*) curves acquired on Dev. 2′ allow to further highlight the differences with the electronic states of Dev. 2. At the extremities of Dev. 2′ (i.e., points p_2_, p_6_ in Fig. 5h), the (d*I*/d*V*)/(*I*/*V*) curves are very similar and show one single peak of occupied DOS centered at ~−1.4 V. The main changes in the (d*I*/d*V*)/(*I*/*V*) curves appear at the central part of Dev 2′ (p_4_) where two peaks of occupied DOS centered at ~−1.3 V and −2.0 V are observed, differently to the one acquired in Dev. 2 at the same position. A careful look at the (d*I*/d*V*)/(*I*/*V*) curves measured at p_3_ and p_5_ in Dev. 2′ identifies also two distinct peaks of occupied DOS centered at −1.2 and −1.8 V showing that the electronic structure of Dev. 2′ is also perturbed along the Si-DB_//_ section of the device (i.e., along the Si-DBs N° 1, 2, and 4). Here, the zig-zag shape of the Si-DB_//_ section made by the Si-DB_2_ displacement in Dev. 2 does not change the intrinsic properties of its Si-DB_//_ section. Indeed, the (d*I*/d*V*)/(*I*/*V*) curves acquired on a fully aligned or a zig-zag 3-Si-DB_//_ exhibit a similar perturbation of the surrounding silicon valence band (Supplementary Fig. 7). Looking at the interactions position p_3_, p_4_, and p_5_ in Dev. 2′ reveal another major modification of its electronic structure where one can observe that the unoccupied (d*I*/d*V*)/(*I*/*V*) signals in the range 0.9–1.5 V have a significantly lower intensity compared to Dev. 2 (see red arrows in Fig. 5f, h) indicating that the lateral electronic coupling above *E*_F_ is decreased in Dev. 2′ compared to Dev. 2, especially along the branches p_3_ and p_5_ of the device.

**Fig. 6:**
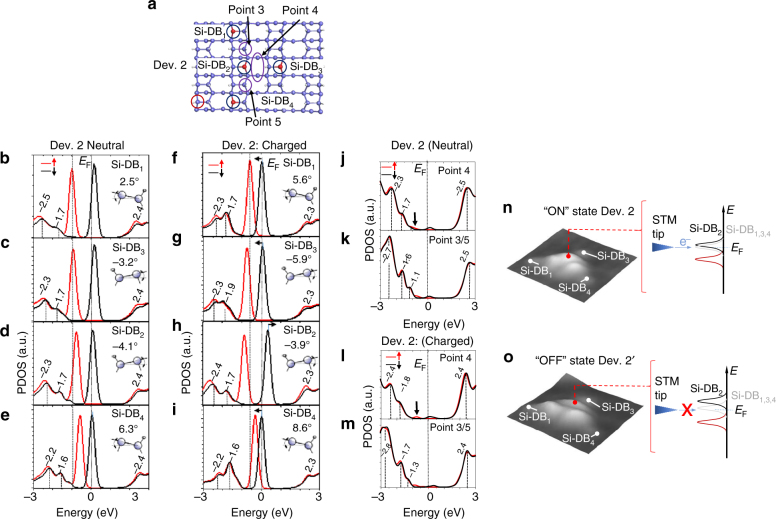
Analysis of the neutral and charged states of Dev. 2. **a** Ball and stick sketch of the four Si-DB structures of Dev. 2. The blue and white balls are Si and H atoms, respectively. **b**–**e** PDOS curves of the spin-up (red) and spin-down (black) states calculated at the four Si-DB locations on Dev. 2 (neutral state). **f**–**i** PDOS curves of the spin-up (red) and spin-down (black) states calculated at the four Si-DB locations of Dev. 2 in its charged state Dev. 2′. The tilt angles of the silicon dimers holding the Si-DBs are indicated in the insets. **j**–**m** PDOS curves of the spin-up (red) and spin-down (black) states calculated at the coupling positions 4, 3, and 5 in the neutral state Dev. 2 in **j** and **k** and the charged state Dev. 2′ in **l** and **m**. **n**, **o** Three-dimensional STM topographies of the Dev. 2 in its ON (**n**) and OFF (**o**) states

Reading the (d*I*/d*V*)/(*I*/*V*) curves shown in Fig. 5h clearly confirms that the reversible switching between Dev. 2 and Dev. 2′ is related to a change in its electronic structure. Considering that one state of the device is triggered with an excitation at positive bias (i.e., injection of tunnel electrons in the empty states) and that the second switching state is obtained by applying a negative bias (i.e., injection of holes in the valence band of the structure) shows that the device presented in Fig. 5d can store, in a stationary state, additional electronic charge. This process that has been so far only performed on a single Si-DB on a p-type doped silicon sample^16^ is shown to be reversible in Dev. 2/2′ on an n-type doped silicon sample. The DFT simulations performed on Dev. 2 confirm this effect (Fig. 6a). The ensuing PDOS curves calculated at each Si-DBs of Dev. 2 show that all the partially charged spin-down states of the Si-DBs pin the Fermi level of the Si(100):H surface (Fig. 6b–e). This shows that all the Si-DB states that cross the surface Fermi level are delocalized over the 2D structure and thus can be used as lateral electronic transport channels from each extremity of the device (i.e., the Si-DBs N° 1, 3, or 4) when the energy of these states are kept within *E*_F_, i.e., at very low bias. This effect can be indirectly probed at higher biases in Dev. 2 when the electronic transport arising from the unoccupied states is strong at the interaction points p_3_ and p_5_ (doted rectangle in Fig. 5f).

The computed occupied bands in the PDOS curves series of Dev. 2 (Fig. 6b–e) show two main delocalized peaks (−2.3 and −1.7 eV) indicating that the device has a homogeneous valence band as observed experimentally. Coherently, the same PDOS peaks can also be observed at point 4, i.e., in between the Si-DB_2_ and Si-DB_3_ (Fig. 6j). The two lateral positions at points 3 and 5 exhibit different valence PDOS peaks (−1.1, −1.6, and −2.7 eV in Fig. 6k), reproducing the trends observed in Fig. 5f where the *σ* band peaks energies in the (d*I*/d*V*)/(*I*/*V*) curves at the points p_3_ and p_5_ are slightly shifted to higher energies. It is interesting to observe that the electronic structure of the two Si-DB_//_ pairs in Dev. 2 do not exhibit similar spin-up state energies (Fig. 6b–e), whereas this is the case for a single 3-Si-DB_//_ having a zig-zag shape (Supplementary Fig. 7g). This effect results of the specific 2D combination of the two Si-DB_//_ pairs that couple to one Si-DB⊥ pair.

When an additional electron is stored in Dev. 2′, the extra charge is shown to be delocalized over the three extreme Si-DBs of the structure (Fig. 6f, g, and i) resulting in the energy shift of the spin-down PDOS peaks of the Si-DBs N° 1, 3, and 4 toward *E*_F_. Inversely, the Si-DB_2_ (i.e., the central part of Dev. 2′) is now in a neutral state since its PDOS spin-down state peak is entirely above *E*_F_ (Fig. 6h), effect that extends to the point p_4_ (Fig. 6l) indicating that the two Si-DB pairs of the horizontal branch of Dev. 2 are less hybridized and have thus strongly reduced conducting channels at *E*_F_. The stored extra charge in Dev. 2′ is shown to be compensated by an increase of the tilt angle of the silicon dimers holding the Si-DB_1_, Si-DB_3_, and Si-DB_4_, whereas the tilt angle of the central silicon dimer (Si-DB_2_) coherently decreases (see the insets in Fig. 6f–i). Note that the PDOS curves in Fig. 6m in the range 0 to −2 eV at the positions 3 and 5 (peaks at −1.7 and -1.3 eV) reproduce the experimental (d*I*/d*V*)/(*I*/*V*) DOS peaks measured at p_3_ and p_5_ (−1.8 and −1.2 V) in Fig. 5h. Our results demonstrate that the electronic state switching between Dev. 2 and Dev. 2′ arises from an additional charge that induces a complete reorganization of its electronic structure. Our DFT simulations also fit with the experimental STM topographies changes observed between Dev. 2 and Dev. 2′ and in particular the spatial extension of the central coupling protrusion between Si-DB_2_ and Si-DB_3_ (Supplementary Fig. 10). It is important to underline that neither the 3-Si-DB_//_ nor the Dev. 1 structures could be switched electronically as shown for Dev. 2 and Dev. 2′ underlining the fact that the electronic properties of Dev. 2 arise from a specific anisotropic interactions between the Si-DBs.

## Discussion

The ensemble of these experimental and theoretical results shows clear evidences that the “Y”-shaped Si-DB structure Dev. 2 can reversibly switch between two electronic states inducing specific electronic modifications to the Si-DB device. This suggests that the central part of the device (i.e., Si-DB_2_ and the interaction point 4) has a function similar to an atomic-scale pinch area that opens (Dev. 2′) or closes (Dev. 2) a local gap within the Si-DB_2_ position. The electronic configuration of Dev. 2′ will strongly reduce the lateral electronic transport at *E*_F_ from any branch of the device and in particular from Si-DB_1_ to Si-DB_4_, along the [110] direction because of the local gap at Si-DB_2_ and thus gives to the devices Dev. 2/2′ an ON or OFF function that can be triggered by the tunnel electrons (Fig. 6n, o). Here, the fact that the hybridized Si-DB states energies are localized within the gap state of the Si(100):H surface provides adequate conditions to reduce current leakage due to the weak electronic coupling with the substrate^37,38^. In these conditions, the running bias of the device may be chosen such that the conducting channel with the gap state is minimized to optimize the lateral electronic conductance. Hence, working at very low temperature (9 K) stabilizes the tilt angle of the Si dimers that holds the Si-DBs and allows the exploitation of these anisotropic properties. Unfortunately, such device cannot yet be fully tested with real contacts due to inexistent tool that can be adapted to its size such as, for example, a multi-probe STM.

This work shows that the anisotropic interactions of the Si-DB QD on the Si(100):H surface enable the fabrication of QD structures at the quantum level in which original electronic processes can be studied and exploited for the realization of 2D devices such as, charge qubits^39^, quantum Hamiltonian computers^31^, functionalized nanoscale wire^40,41^, or to create various functionalized molecular anchoring bridges^19^.

## Methods

### Experimental methods

The experiments are performed with a low-temperature (9 K) STM from CREATEC running under ultra-high vacuum (below 2 × 10^−11^ torr). The samples used are n-type doped (As, concentration of 5 × 10^19^ atoms per cm^3^, *ρ*~5 mΩ.cm) made of very pure crystalline silicon (ITME, Institute of Electronic Material Technology) cut along the (100) plane. The Si(100):H surface are prepared under UHV (Ultra High Vaccum) via two steps. The first step consists in cleaning the silicon surface by removing the oxide layer. Then, its reconstruction is obtained through a repeated cycle of short heating at 1100 °C during 1–3 s followed by a longer heating lasting for ~2 min in the range 950–650 °C. After the surface reconstruction, the sample is exposed to dihydrogen in front of a hot (~1500 °C) tungsten filament that plays the role of an electron gun to excite the hydrogen atoms^42^. Our measurements have been repeated on three different samples and STM tips that have been rigorously prepared in the same conditions to prevent any measurement artifacts^8^. The STM tips are formed in air via an electrochemical etching process in a diluted solution of NaOH. Our tip preparation method allows to control the radius of curvature of the apex in a range varying between 10 and 50 nm^27^. The STM tips are then cleaned under vacuum via electrons bombardment before their transfer inside the STM. The Si-DBs are created in situ with the STM tip by applying a negative pulse on the desired Si–H bond. This process is well controlled and allows very local reproducible desorptions^15,43^. The scanning tunneling spectroscopy (STS) curves are extracted from a lock-in amplifier. A second lock-in amplifier is working simultaneously and synchronized with the first one. The (d*I*/d*V*)/(*I*/*V*) signals extracted from both lock-in amplifiers are compared after each measurements to avoid glitch or artifacts. The sample bias modulation during the STS measurements is fixed at a frequency of 732 Hz for both lock-in amplifiers with an amplitude of 10–30 mV. The tunnel current is converted into a voltage signal outside the UHV chamber with an intrinsic noise of ~1–5 mV giving a robust average signal-to-noise ratio on the (d*I*/d*V*)/(*I*/*V*) signal. These experimental conditions provide a very good accuracy and repeatability to our measurements. Each (d*I*/d*V*)/(*I*/*V*) curve is acquired at various STM tip heights in particular to estimate the possible energy shifts of the DOS peaks due to STM tip-induced band -bending effects. The ensuing (d*I*/d*V*)/(*I*/*V*) data presented in this article are representative of reproducible sets of measurements. The STM topographies of the different structures observed in this work have been realized several times on the used samples on different Si(100):H terraces orientations in order to avoid micro-tip shape effects and possible local variations of subsurface dopants concentration. Note that the three different STM tips used during our experiments provide the same observations when the Si-DB_//-⊥_ are created on each type of the Si(100):H terraces (S_A_ or S_B_), which is ruling out any possible anisotropic effect due to a specific STM apex tip shape^15^.

### Theoretical methods

The ab initio SIESTA code running spin-polarized DFT calculations^44^ is used to describe the structural and electronic properties of a hydrogenated silicon slab. The slab is constructed from a Si(100):H surface unit cell, which size (46.32 × 77.21 × 30 Å^3^) counts a total number of 1680 atoms (i.e., 959 Si, 720 H, and 1 As atoms) without dangling bonds. H atoms are used on the back side of the slab in order to neutralize the Si back-bond states of the last Si layer providing a total slab thickness of 6.56 Å (i.e., four atomic Si layers + two H layers). Here the As atom is located at the third silicon layer (from the 2 × 1 surface), far from the Si-DBs to get rid of any influence of its position on the electronic structure^32^ (a detailed description of the slab is given in the Supplementary Fig. 11). The generalized gradient approximation within the Perdew Burke Ernzerhof is used to describe the exchange correlation energy^45^. Troullier Martins type pseudopotentials^46^ and single zeta polarized function basis set is used to describe the valence state wavefunction. The conjugate gradient method is employed to perform the geometry optimization of the structure by using the self-consistency mixing rate of 0.1, a maximum force tolerance of 0.02 eV / Å and a mesh cutoff of 300 Ry (the variations of these parameters showed a very low perturbation of the total energies by <0.1%). To sample the Brillouin zone, a set of nine Monkhorst–Pack^47^ special k-points was used. The self-consistent cycles were stopped when the variations of the total energy per unit cell and band structure energy were both <10^-4^ eV for each of the studied Si-DB structures (Si-DB_//_, Si-DB_⊥_, and the four DB structures Dev. 2 and Dev. 2′). All pertinent information such as the partial LDOS and the PDOS are obtained thereafter from the optimized structures via single-point energy simulations. The DFT simulations of the device Dev. 2 and Dev. 2′ (neutral and charged) are performed by adding (or not) a single charge (i.e., one electron) in the slab that is spreading over the entire surface states. However, to keep the entire system in its neutral electronic state, a compensating background charge is added. For these simulations, the DFT calculations have been performed for two different positions of the As dopant atom in the slab (Supplementary Fig. 11) without showing significant differences in the presented results. The main role of the As dopant atom is thus to provide extra charge to the empty state of the Si-DBs. A comparison of the As atom electronic structure with and without Si-DBs is provided in the Supplementary Fig. 12 and Note 8.

### Data availability

The data that support the findings of this study are available from the corresponding author upon reasonable request.

## Electronic supplementary material

Supplementary Information

## References

[1] Fölsch S, Martinez-Blanco J, Yang J, Kanisawa K, Erwin SC (2014). Quantum dots with single-atom precision. Nat. Nano.

[2] Usman M (2016). Spatial metrology of dopants in silicon with exact lattice precision. Nat. Nano.

[3] Pan Y, Yang J, Erwin SC, Kanisawa K, Fölsch S (2015). Reconfigurable quantum-dot molecules created by atom manipulation. Phys. Rev. Lett..

[4] Kitchen D, Richardella A, Tang JM, Flatté ME, Yazdani A (2006). Atom-by-atom substitution of Mn in GaAs and visualization of their hole-mediated interactions. Nature.

[5] Nowakowska S (2016). Controlling electronic states in an atomically precise array of quantum boxes. Small.

[6] Stokbro K (1998). STM-induced hydrogen desorption via a hole resonance. Phys. Rev. Lett..

[7] Kleshchonok A, Gutierrez R, Joachim Ch, Cuniberti G (2015). Quantum interference based Boolean gates in dangling bond loops on Si(100):H surfaces. Sci. Rep..

[8] Taucer M (2014). Single-electron dynamics of an atomic silicon quantum dot on the H-Si(100)-(2×1) surface. Phys. Rev. Lett..

[9] Bellec A (2009). Electronic properties of the n-doped hydrogenated silicon (100) surface and dehydrogenated structures at 5K. Phys. Rev. B.

[10] Naydenov B, Boland JJ (2013). Engineering the electronic structure of surface dangling bond nanowires of different size and dimensionality. Nanotechnology.

[11] Godlewski S (2015). Dynamical behavior of a dangling bond dimer on a hydrogenated semiconductor: Ge(001):H. Phys. Rev. B.

[12] Nguyen TH (2010). Coulomb energy determination of a single Si dangling bond. Phys. Rev. Lett..

[13] Bellec A, Ample F, Riedel D, Dujardin G, Joachim Ch (2008). Imaging molecular orbitals by scanning tunneling microscopy on a passivated semiconductor. Nano Lett..

[14] Livadaru L, Pitters J, Taucer M, Wolkow RA (2011). Theory of nonequilibrium single-electron dynamics in STM imaging of dangling bonds on a hydrogenated silicon surface. Phys. Rev. B.

[15] Bellec A (2010). Nonlocal activation of a bistable atom through a surface state charge-transfer process on Si(100)-(2×1):H. Phys. Rev. Lett..

[16] Bellec A (2013). Reversible charge storage in a single silicon atom. Phys. Rev. B.

[17] Haider MB (2009). Controlled coupling and occupation of silicon atomic quantum dots at room temperature. Phys. Rev. Lett..

[18] Schofield SR (2013). Quantum engineering at the silicon surface using dangling bonds. Nat. Commun..

[19] Godlewski S (2016). Single-molecule rotational switch on a dangling bond dimer bearing. ACS Nano.

[20] Labidi H (2015). Scanning tunneling spectroscopy reveals a silicon dangling bond charge state transition. New J. Phys..

[21] Yengui M, Pinto HP, Leszczynski J, Riedel D (2015). Atomic scale study of corrugating and anticorrugating states on the bare Si(100) surface. J. Phys. Condens. Matter.

[22] Okada H, Fujimoto Y, Endo K, Hirose K, Mori Y (2001). Detailed analysis of scanning tunneling microscopy images of the Si(100) reconstructed surface with buckled dimers. Phys. Rev. B.

[23] Ihm J, Lee DH, Joannopoulos JD, Xiong JJ (1983). Structural phase diagrams for the surface of a solid: a total-energy, renormalization-group approach. Phys. Rev. Lett..

[24] Pennec Y, Horn von Hoegen M, Zhu X, Fortin DC, Freeman MR (2006). Dynamics of an ising chain under local excitation: a scanning tunneling microscopy study of Si(100) dimer rows at 5K. Phys. Rev. Lett..

[25] Lee JY, Cho JH, Zhang Z (2009). Quantum size effects in competing charge and spin orderings of dangling bond wires on Si(001). Phys. Rev. B.

[26] Labidi H, Kantorovich L, Riedel D (2012). Atomic-scale control of hydrogen bonding on a bare Si(100)-2×1 surface. Phys. Rev. B.

[27] Riedel D, Delattre R, Borisov AG, Teperik TV (2010). A scanning tunneling microscope as a tunable nanoantenna for atomic scale control of optical-field enhancement. Nano Lett..

[28] Bayat A, Creffield CE, Jefferson JH, Pepper M, Bose S (2015). Quantum dot spin cellular automata for realizing a quantum processor. Semicond. Sci. Technol..

[29] Ushakov AV, Streltsov SV, Khomskii DI (2011). Crystal field splitting in correlated systems with negative charge-transfer gap. J. Phys. Condens. Matter.

[30] Fuechsle M (2012). A single-atom transistor. Nat. Nano.

[31] Kolmer M (2015). Realization of a quantum Hamiltonian Boolean logic gate on the Si(001):H surface. Nanoscale.

[32] Kepenekian M, Robles R, Rurali R, Lorente N (2014). Spin transport in dangling-bond wires on doped H-passivated Si(100). Nanotechnology.

[33] Hitosugi T (1999). Jahn-Teller distortion in dangling-bond linear chains fabricated on a hydrogen-terminated Si(100)- 2×1 surface. Phys. Rev. Lett..

[34] McEllistrem M, Haase G, Chen D, Hamers R (1993). Electrostatic sample-tip interactions in the scanning tunneling microscope. Phys. Rev. Lett..

[35] Labidi H, Sonnet Ph, Riedel D (2013). Electronic control of the tip-induced hopping of an hexaphenyl-benzene molecule physisorbed on a bare Si(100) surface at 9K. J. Phys. Chem. C.

[36] Yengui M, Riedel D (2015). Evidence of low Schottky barrier effects and the role of gap states in the electronic transport through individual CoSi_2_ silicide nanoislands at low temperature (9K). J. Phys. Chem. C.

[37] Kepenekian M, Robles R, Joachim Ch, Lorente N (2013). Leakage current in atomic size surface interconnects. Appl. Phys. Lett..

[38] Bohoul S, Shi Q, Wolkow RA, Guo H (2017). Quantum transport in gated Dangling-bond atomic wires. Nano Lett..

[39] Livadaru L (2010). Dangling-bond charge qubit on a silicon surface. New J. Phys..

[40] Engelund M (2016). Search for metallic dangling-bond wire on n-doped H-passivated semiconductor surfaces. J. Phys. Chem. C.

[41] Kepenekian M, Robles R, Joachim Ch, Lorente N (2013). Surface-state engineering for interconnects on H-passivated Si(100). Nano Lett..

[42] Riedel, D. in *Advances in Atom and Single Molecule Machine Series*, Surface hydrogenation of the Si(100)-2×1 and electronic properties of silicon dangling bonds on the Si(100):H (Springer, 2017).

[43] Martin M (2006). Mastering the molecular dynamics of a bistable molecule by single atom manipulation. Phys. Rev. Lett..

[44] Ordejón P, Artacho E, Soler MJ (1996). Self-consistent order-N density-functional calculations for very large systems. Phys. Rev. B.

[45] Perdew JP, Burke K, Ernzerhof M (1996). Generalized gradient approximation made simple. Phys. Rev. Lett..

[46] Troullier N, Martins JL (1991). Efficient pseudopotentials for plane-wave calculations. Phys. Rev. B.

[47] Monkhorst HJ, Pack D (1976). Special points for Brillouin-zone integrations. Phys. Rev. B.

