# Supplementary material for: A two-dimensional ON/OFF switching device based on anisotropic interactions of atomic quantum dots on Si(100):H
Source: Nat Commun. 2017 Dec 20;8:2211. doi: 10.1038/s41467-017-02377-4 (PMC5738427; doi:10.1038/s41467-017-02377-4)
Supplement: Supplementary file 1 — Supplementary Information [file 41467_2017_2377_MOESM1_ESM.pdf]

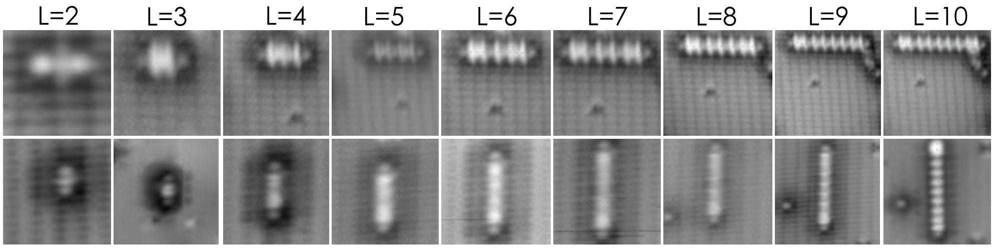

Supplementary Figure 1: Comparison of the unoccupied states STM topographies as a function of the Si-DB lengths and directions along the $[1\bar{1}0]$ and [110] axis of the Si(100):H surface. STM topographies series (V= 1.8 V, I = 25 pA) of increasing lengths (L = 2 to L = 10, L is the number of Si-DB in the line) oriented across and along the direction of the silicon dimer rows.

Supplementary Note 1: The contribution of the lateral electronic coupling through the spin-down empty states may induce electronic standing waves along the Si-DB$_{//-\perp}$, revealing nodes and anti-nodes of charge density in the STM topographies (Figs. 1 for L=3 to L= 8)[1,2,3]. This effect is only clearly observed experimentally for Si-DB lines having a length longer than 3 Si-DBs (i.e. > ~ 16 Å).

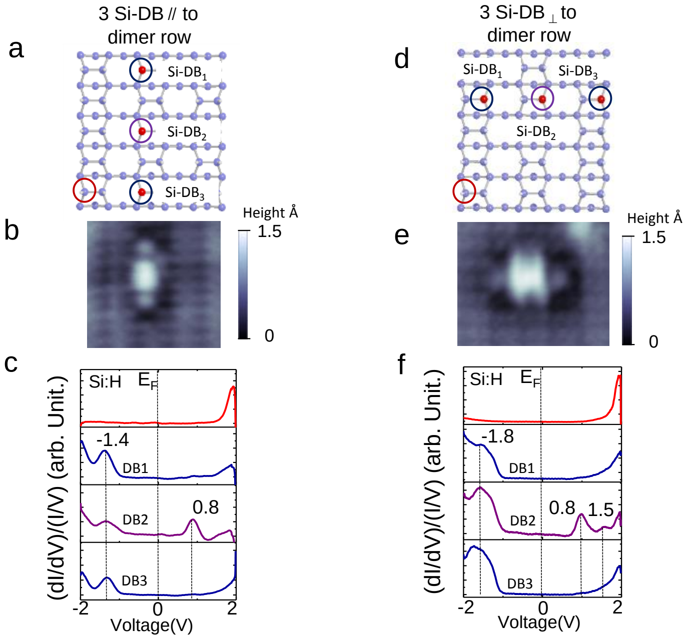

Supplementary Figure 2: Topographic and electronic aspects of the anisotropic Si-DB interactions for a 3 Si-DB line. (a) and (d) ball and stick sketches of the Si-DB$_{//}$ and Si-DB$_{\perp}$ lines made of 3 Si-DBs oriented parallel (a) or perpendicular (d) to the [110] direction of the silicon lattice. The blue, purple and red circles indicate the atom on which the dI/dV measurements are done. (b) and (e) (31 x 39 Å^2) and (38.7 x 27 Å^2) STM topographies (V = +1.7 V, I = 33 pA) of the 3 Si-DBs lines separated by a fully hydrogenated silicon dimer. (c) and (f) normalized (dI/dV)/(I/V) curves measured on the corresponding Si-DB lines. The (dI/dV)/(I/V) curves on the Si:H, the Si-DB positions, or central to the Si-DB line are red, blue and purple, respectively.

Supplementary Note 2: Figs. 2b and 2e show clear evidences that the unoccupied STM topographies of the Si-DB$_{//}$ and Si-DB$_{\perp}$ lines exhibit two different spatial distributions of charge density. The ensuing bright protrusions, signature of the Si-DB coupling is mainly located at the central Si-DB atom for the Si-DB$_{//}$ line (Fig. 2b) while the charge density extends in between the Si-DBs at the Si-DB$_{\perp}$ line (Fig. 2e). The (d*I*/d*V*)/(*I*/*V*) curves acquired along the Si-DB$_{//}$ line show similar trend observed for the case of a 2 Si-DB$_{//}$ line (Fig. 1 of the main text) with one peak of occupied DOS at -1.4 V and a single unoccupied DOS peak at 0.8 V. The (d*I*/d*V*)/(*I*/*V*) curves acquired along the Si-DB$_{\perp}$ line show the same occupied DOS peak as observed in the case of a 2 Si-DB$_{\perp}$ line whereas the unoccupied part of the spectrum indicate that two DOS peaks (0.8 V and 1.5 V) can be observed.

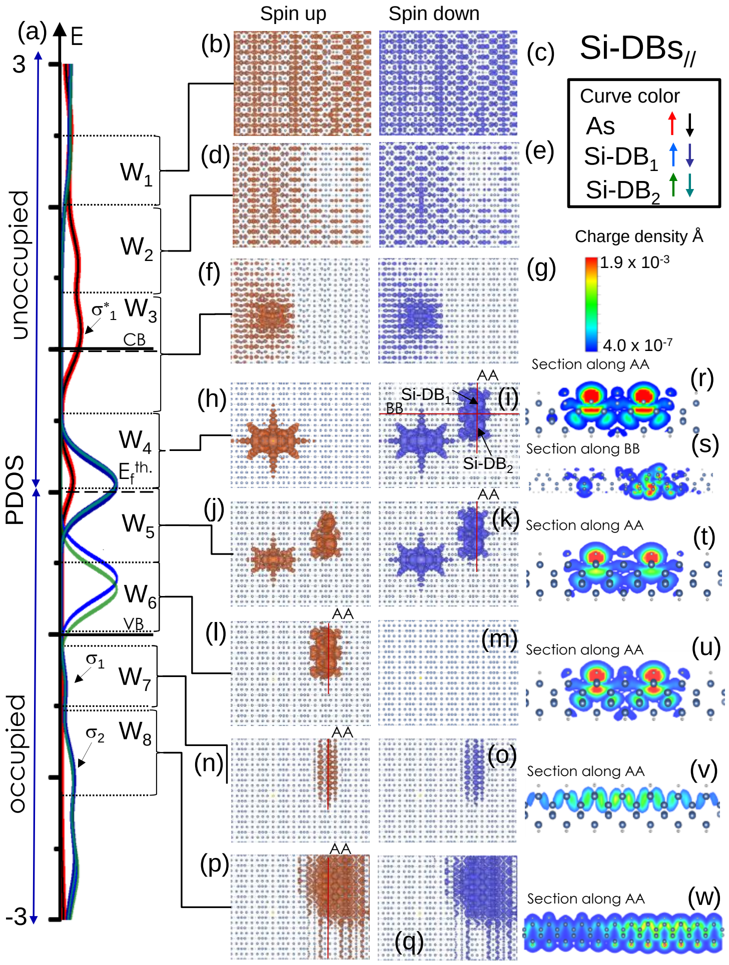

Supplementary Figure 3: Evolution of the LDOS isodensities distributions of the Si-DB$_{//}$ line as a function of the energy. (a) Calculated PDOS curves at the Si-DBs for the 2 Si-DB$_{//}$ and the As atom (recall from Fig. 2). The presented energy range is divided in 8 energy windows W_1 to W_8. (b) –(q) spin-up and spin-down partial LDOS isodensities distributions over the silicon slab for the eight energy windows. (r) – (w) cross sectional plot of the partial LDOS for the energy windows W_4 to W_8 along the AA or BB axis defined in the ensuing 2D plots.

Supplementary Note 3: The additional LDOS distributions shown in Figs. 3n to 3q are related to the occupied σ orbitals (W_7 and W_8) of the Si(100):H surface delocalized through the subsurface Si-Si back-bonds network. We can note the clear difference between the LDOS distribution direction and depth inside the slab related to the σ_1 band (W_7) in the sectional drawing of Fig. 3v, which is particularly extended along the silicon dimer row ([110] direction) compared to the σ_2 band (Fig. 3w) that shows a deeper spatial distribution of LDOS in both directions (i.e. [110] and [1-10]). This observation is strongly coherent with the detection of a DOS peak at -1.4 V in the (d*I*/d*V*)/(*I*/*V*) curves acquired at the

Si-DB// lines (i.e. in the same energy range as the window W_7). The σ^* bands of the Si-Si back-bonds network at the Si(100):H surface (W_2 and W_3) are depicted in Figs. 3d to 3g and show a strong contribution of the As atom at the first unoccupied PDOS peak (PDOS peak at 1.1 eV in W_3) and a more delocalized distribution of LDOS observed in the energy window W_2 (PDOS peak at 1.6 eV in W_2). Here we also remark the LDOS depletion in the area of the Si-DB// line at the energy window W_2 (Figs. 3d and 3e) that can be related to the dark halo observed experimentally surrounding the STM topographies.

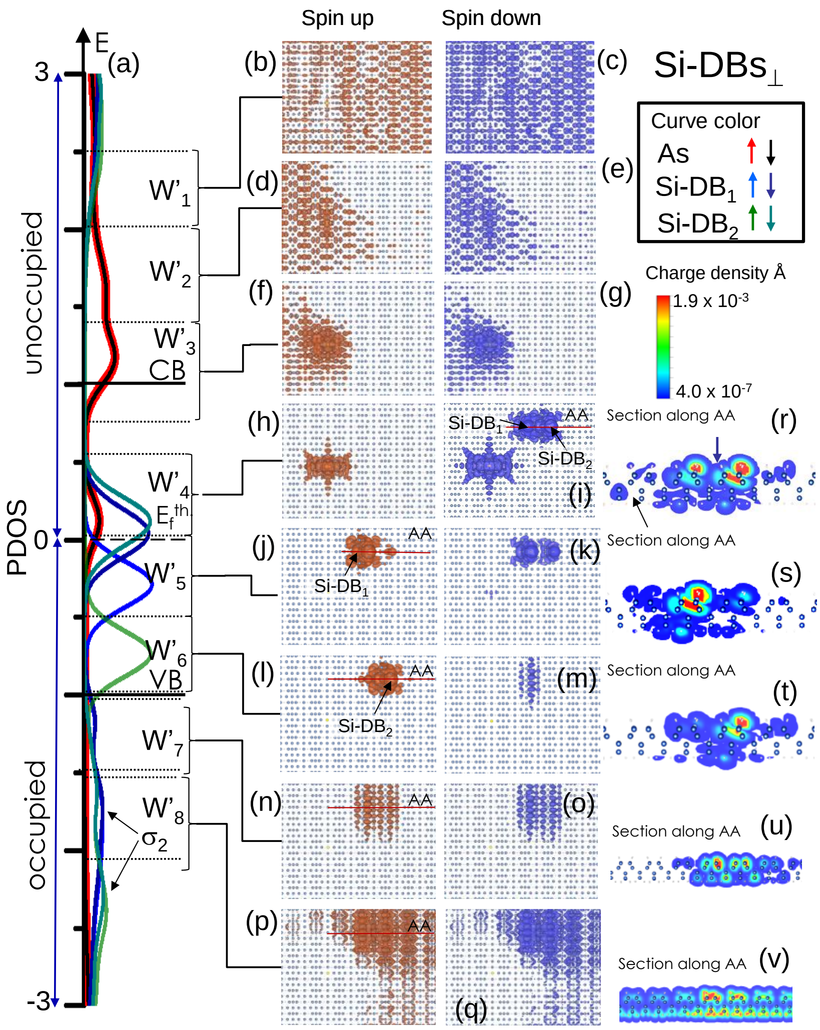

Supplementary Figure 4: Evolution of the LDOS isodensities distributions of the Si-DB⊥ line as a function of the energy. (a) Calculated PDOS curves at the Si-DBs for the 2 Si-DB⊥ and the As atom (recall from Fig. 2). The presented energy range is divided in 8 energy windows W'_1 to W'_8. (b) –(q) spin-up and spin-down partial local density of states distributions over the silicon slab for the eight energy windows. (r) – (v) cross sectional plot of the partial local density of state for the energy windows W'_4 to W'_8 along the AA axis defined in the ensuing 2D plots.

Supplementary Note 4: The LDOS distributions for Si-DB⊥ presented in Fig. 4 at various energy windows (W'_1 to W'_8) can be correlated with the calculated PDOS curves recalled in Fig. 4a. The LDOS distribution in the valence bands energies windows W'_7 and W'_8 shows good similarities with the one observed previously for the Si-DB// except that they are both delocalized in the two [110] and [1-10] directions (Figs. 4n to 4q) and are centered at higher energies (-1.2 V and -1.7 V). This also reveals how

the interaction between the Si-DB acts differently on the valence bands energies whether the Si-DB are involved in Si-DB$_{\perp}$ or Si-DB$_{//}$ lines. The analysis of the LDOS distribution in the conduction band (Figs. 4b to 4g) reveals the possible hybridization of the unoccupied states surrounding the As dopant atom with the unoccupied LDOS related to the Si-DB$_{\perp}$ line when imaged with the STM tip. This result further confirms that the area located within the silicon dangling bonds exhibits a LDOS depletion in the energy windows related to the σ* bands of the Si(100):H surface.

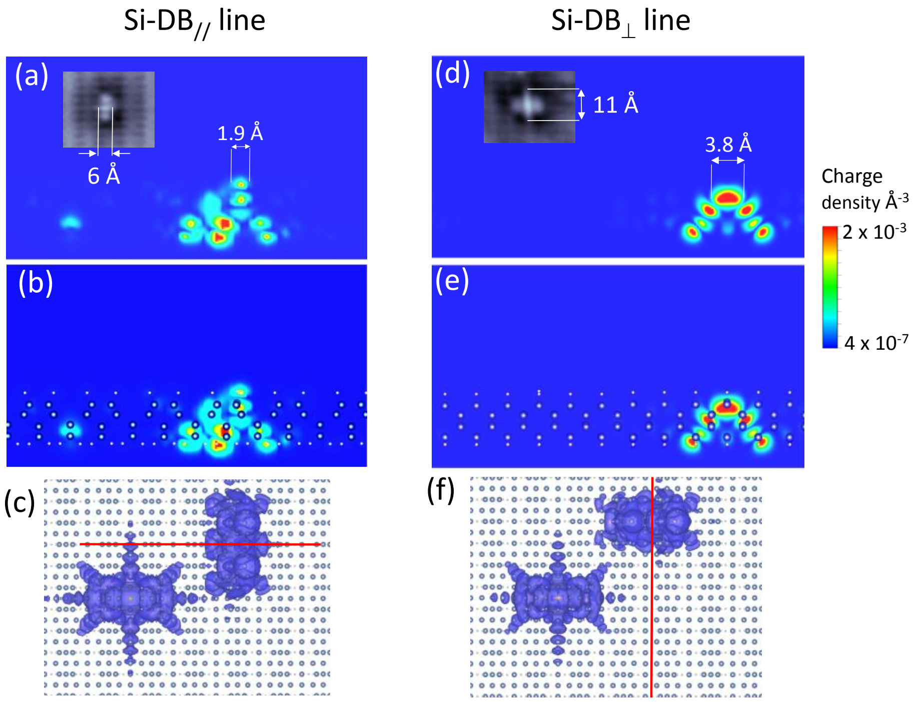

Supplementary Figure 5: Comparison of the cross-sectional LDOS distributions in the protrusion areas for the Si-DB$_{//}$ and Si-DB$_{\perp}$ lines. (a) and (b) are two dimensional LDOS cross sectional distributions at the central part of a Si-DB$_{//}$ line. (d) and (e) are two dimensional LDOS cross sectional distributions at the central part of a Si-DB$_{\perp}$ line. (c) and (f) are ball and stick representation of the selected portion of silicon slab with the ensuing LDOS isosurfaces taken from Supplementary Figs. 3i and 4i in which the red lines indicate where the plans of the cross sectional distributions are located. The insert in the panels 5a and 5d are examples of STM topographies of the corresponding Si-DB lines protrusion for each cases.

Supplementary Note 5: Considering the fact that the unoccupied state STM topographies of the Si-DB lines result of a tunnel electrons transport through empty states at the surface (Si-DBs) and other states via the subsurface (As atom) mixed with lateral electronic coupling, the size of the protrusion estimated from the STM topographies cannot be directly compared with the one computed in the LDOS distributions. However, the ratio of their sizes can be. Here, the ratio between the measured sizes of the protrusion area worth 6 /11 = 0.545 which is in good agreement with the ratio of the computed sizes which is 1.9/3.8 = 0.5.

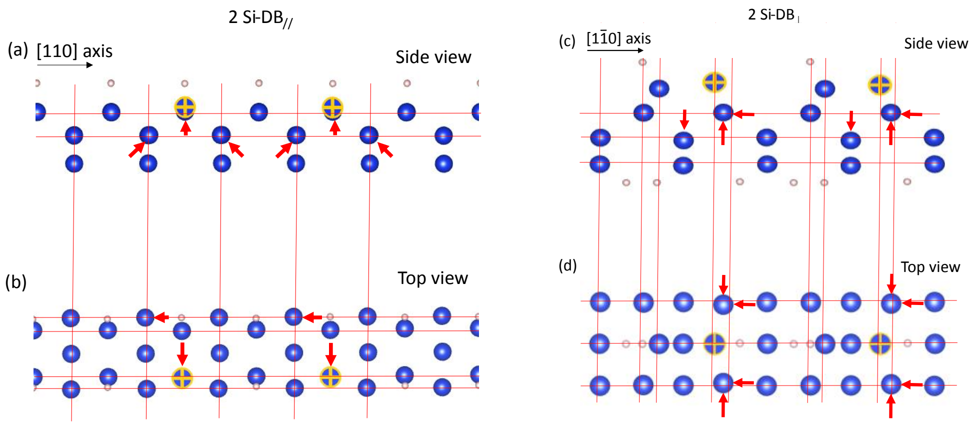

Supplementary Figure 6: Details of the Jahn-Teller silicon lattice distortions for Si-DB$_{//}$ and Si-DB$_{\perp}$. (a) and (b) Ball and stick sketches of the silicon lattice surrounding a Si-DB$_{//}$ line for the side (a) and top (b) views. (c) and (d) Ball and stick sketches of the silicon lattice surrounding a Si-DB$_{\perp}$ line for the side (c) and top (d) views. The Si-DB atoms are highlighted with crossed yellow circle. The red arrows indicate the directions of the Si atoms displacement due to the Jahn-Teller distortion in the vicinity of the Si-DB compared to the averaged position of Si atoms of the fully hydrogenated surface.

Supplementary Note 6: In the Si-DB$_{//}$ line, the Jahn-Teller distortions induce lattice modifications at the first and second atomic silicon layer (Fig. 6a and 6b) involving a local reorganization of the σ_1 band in the Γ-J' direction of the surface Brillouin zone[4] (see Figs. 3n and 3o). This band is lying at energies near the valence band edge and leads, as observed in Figs. 2c and 2e of the main text, to an efficient Si-DB coupling due to degenerated spin-up and spin-down states levels. In the case of the Si-DB$_{\perp}$ line, the lattice distortion is more complex and implicates a deeper structural change (Figs. 6c and 6d). The silicon atoms of the second and third layers are moved and hence modify locally the Si-Si back-bonds which valence band spreads in various crystallographic directions. A subsequent charge ordering is necessary to reorganize the local electronic structure leading to the observed Si-DB$_{\perp}$ hybridization through the grooves that separates two silicon dimer rows. This process involves mainly the occupied σ_2 band in both the Γ-J' and Γ-J directions of the surface Brillouin zone (Supplementary Figs. 4p and 4q).

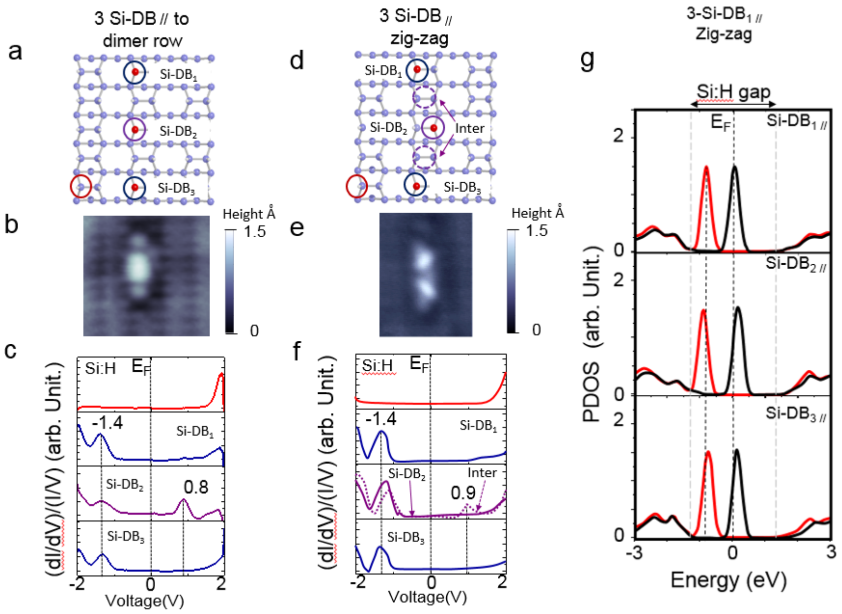

Supplementary Figure 7: Comparison of the electronic structure of a 3 Si-DB$_{//}$ line in the fully aligned or zig-zag shapes. (a) and **(d)** ball and stick sketches of the Si-DB$_{//}$ made of 3 Si-DBs oriented fully aligned (**a**) or in zig-zag (**d**) to the [110] direction of the silicon lattice. The blue, purple and red circles indicate the atom where the dI/dV measurements are done. (**b**) and (**e**) (31 x 39 Å^2) and (27 x 39 Å^2) STM topographies (V = +1.7 V, I = 33-40 pA) of the 3 Si-DBs lines separated by a fully hydrogenated silicon dimer. (**c**) and (**f**) normalized (dI/dV)/(I/V) curves measured on the corresponding Si-DB lines. The (dI/dV)/(I/V) curves on the Si:H, the Si-DB positions, or central to the Si-DB line are red, blue and purple, respectively. (**g**) PDOS curves on the Si-DBs of a 3-Si-DB zig-zag chain as shown in Fig. 7d. The red and black curves represents the spin-up and spin-down states, respectively.

Supplementary Note 7: As long as the Si-DBs are located on the same silicon dimer row, the induced charge ordering created at the coupling part of the Si-DB$_{//}$ line only involve the σ_1 band of the silicon subsurface (second silicon atomic layer, see Fig. 3 panel v). When one Si-DB is misaligned from an initial arrangement as it is the case in Dev. 2 (compared to Dev. 1) for the Si-DB$_{//}$ line section, the set of STS curves show the same typical spectroscopy involving one occupied peak of DOS at -1.4 V (σ_1) and a single peak of unoccupied DOS at ~ 0.9 V. The main difference in these conditions is revealed by the vanishing of the electronic stationary wave in the zig-zag 3-Si-DB$_{//}$ line (Supplementary Fig. 7e) as it appears along the fully aligned 3-Si-DB$_{//}$ (Fig. 7b). However the charge ordering protrusions of the zig-zag 3-Si-DB$_{//}$ line is still observed two-by-two with the same characteristics. Interestingly, the PDOS curves at each Si-DBs of the 3-Si-DB zig-zag line exhibit quasi degenerated spin-up states differently to what is observed in Dev. 2 or Dev. 2'.

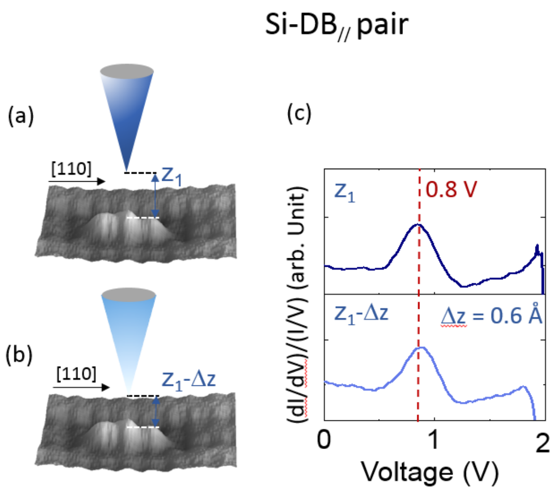

Supplementary Figure 8: Influence of the tip induced band bending on the unoccupied states energy peak in a Si-DB// pair. (a) and (b) STM configuration for an initial STM tip located in the middle of the Si-DB// pair at z_1 and at z_1-Δz, respectively, during the (d*I*/d*V*)/(*I*/*V*) measurements. (c) (d*I*/d*V*)/(*I*/*V*) curves for the measurement at the two considered tip heights (Δ*z* = 0.6 Å).

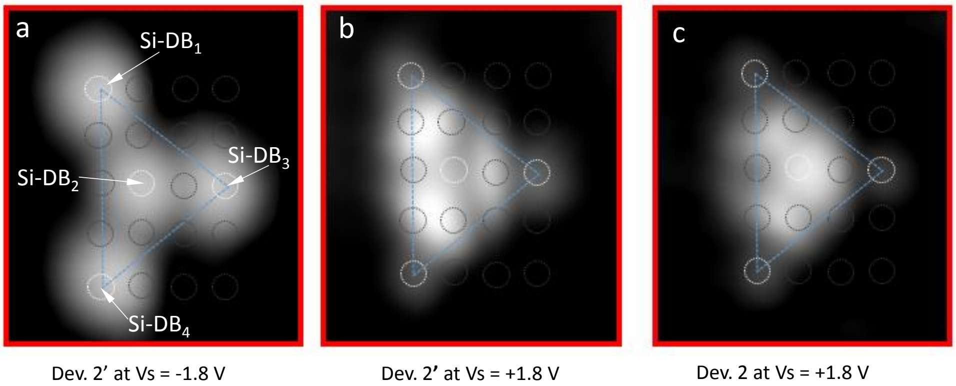

Supplementary Figure 9: Detailed description of the Si-DBs positions between the occupied and unoccupied state STM topographies of the two neutral and charged states of Dev 2 and Dev. 2'. **(a)** (25 x 27 Å²) high contrast occupied state STM topography showing the bright protrusions located at the Si-DBs of the device Dev. 2' in the charged state. The occupied state STM topography of Dev. 2 in the neutral state is the same as in **(a)**. **(b)** and **(c)** (25 x 27 Å²) high contrast unoccupied states STM topographies of Dev. 2' (OFF (charged) state) and Dev. 2 (ON (neutral) state), respectively.

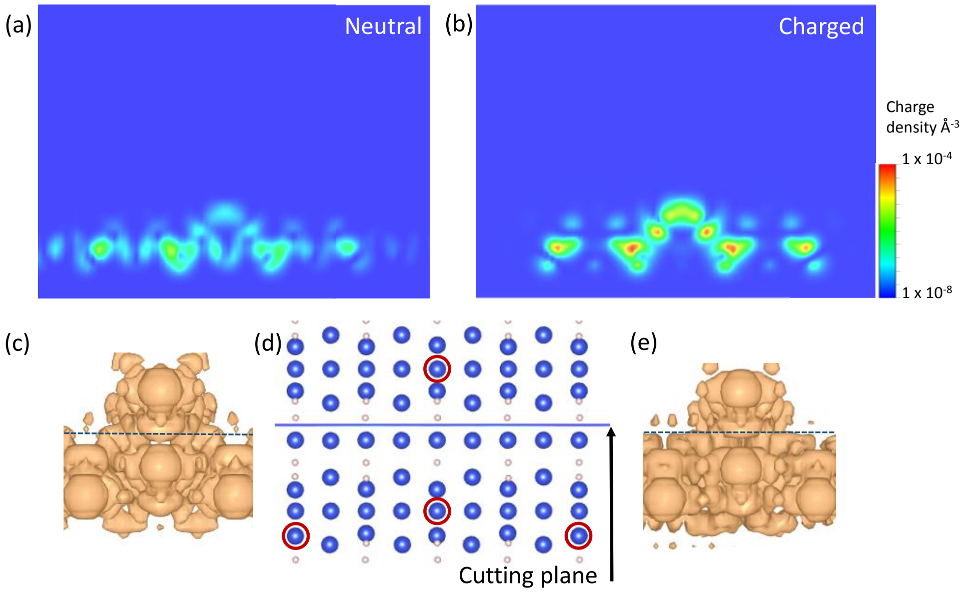

Supplementary Figure 10: Comparison of the cross sectional LDOS distributions between Dev. 2 and Dev. 2'. **(a)** and **(b)** cross sectional LDOS distribution of Dev. 2 (neutral) and Dev. 2' (charged) along the blue dotted lines indicated in **(c)** and **(e).** **(c)** and **(e)** spatial distribution of the LDOS isosurfaces of Dev. 2 and Dev. 2', respectively, in which the location of the cross sectional plans are indicated by a blue dotted line. **(d)** Balls and stick sketch of the silicon surface of the device Dev. 2 or Dev 2'. The silicon atoms are represented by blue balls, the H atoms by white balls and the position of the Si-DB are surrounded by red circles. The blue line in **(d)** recall the position of the chosen sectional drawing plane.

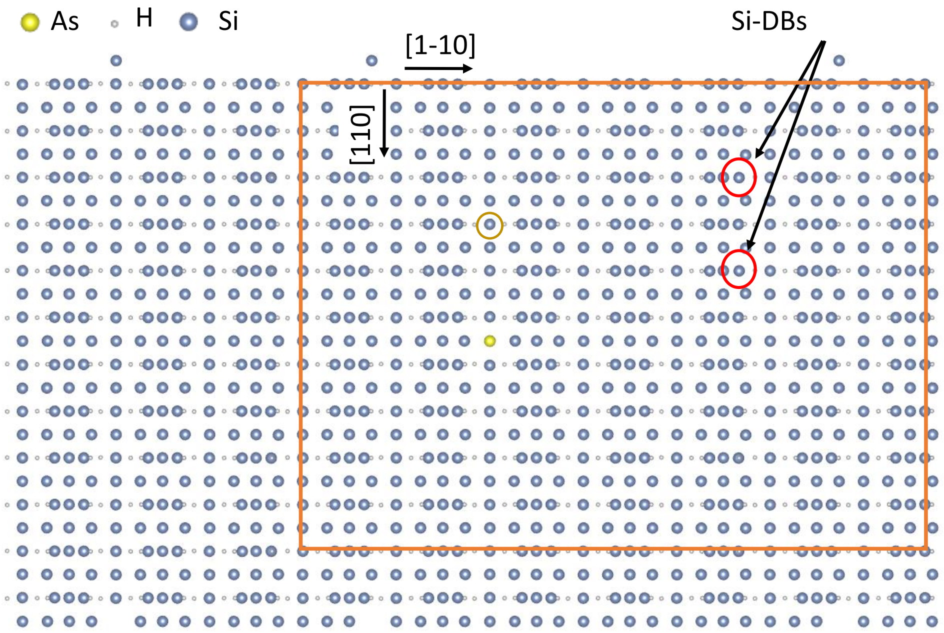

Supplementary Figure 11: Detailed structure of the silicon slab used for the DFT simulations. The silicon slab used for the DFT simulation is formed with 1680 atoms. The red rectangle indicates the size of the area selected to show the LDOS isodensities in Figs. 3 and 4 of the article. Here is an example of a slab used for the DFT simulations of a Si-DB$_{//}$ line. The light brown circle indicate where the second As dopant location has been tested for the DFT simulations.

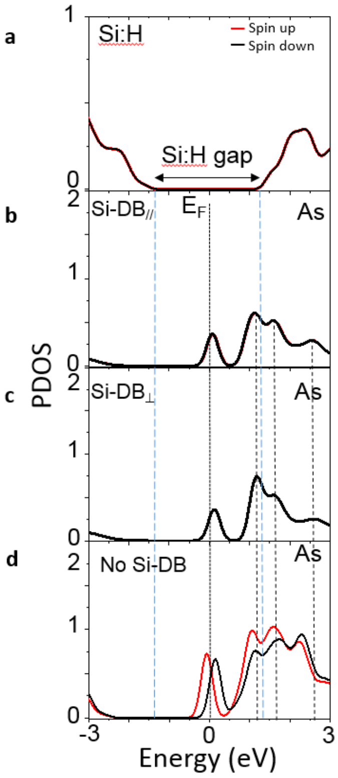

Supplementary Figure 12: Comparison of the PDOS curve at the As dopant atom with or without Si-DBs. (a) PDOS curve at the Si-H atom. **(b), (c)** and **(d)** PDOS curves at the As atom for a slab having a 2-Si-$DB_{//}$ line **(b)**, a 2-Si-$DB_{\perp}$ line **(c)** or fully passivated (no Si-DB) in **(d)**.

Supplementary Note 8: For supplementary Figure 12: The charge of the As atom without Si-DBs is ~ 2.7 times higher than when two Si-DBs are on the surface for both directions. The residual degeneracy shift between the spin-up and spin-down states in the panel d arises from the crystal field created at the Si atoms nearby the As dopant when the residual charge of the As atom slightly spread in its surrounding.

		Valence Band	Band gap	Conduction Band
Experiments	On bare Si(100) from Refs 21.22	σ_2 = -1.8 to -2.5 V<br>σ_1 = -1 to -1.7 V<br>π = - 0.7 to - 1 V	No states in (- 0.9 V to + 0.2 V)	σ_2^* = 1.7 to 2,2 V<br>σ_1^* = 1.1 to 1.7 V<br>π^* = 0.5 to 1.1 V
	On Si(100):H from ref 15	σ = -1 to -2.0 V<br>π = no states	No states in (- 1.1 to + 1.1 V)	σ^* = 1.1 to 1.8 V<br>π^* = no states
	Measures on Si-DB	$-1.8_{\perp}$ V $-1.4_{//}$ V	No states	band (0.5 – 2.0 V)
	Measures in between Si-DBs	$-1.8_{\perp}$ V $-1.4_{//}$ V	No states	$0.8_{//}$ V $1.5_{\perp}$ V
DFT, Si-DB//	DB_1 or DB_2	-2.6 -1.9 -1.3	-0.65 0.07	2.4 to 3.0
DFT, Si-DB//	center DB_1-DB_2 //	-2.8 -2.0 -1.5	-0.65 0.06	2.4 to 3.0
DFT, Si-DB⊥	DB1	-1.8 -1.1	-0.3 0.04	2.4 to 3.0
DFT, Si-DB⊥	DB2	-2.3 -1.6	-0.7 0.1	2.4 to 3.0
DFT, Si-DB⊥	center DB_1-DB_2 ⊥	-2.3 -1.8 -1.2	-0.7 0.1	2.4 to 3.0
DFT	As	No states	0.08	1.1 1.6 2.5
DFT	Si-H	Band (-2.3 to -1.6)	No states	1.5 2.1 2.7

(the energies are relative to E-E_F and expressed in eV)

Supplementary Table 1: Comparative table of the experimental DOS and simulated PDOS peaks on the Si(100) and Si(100):H surfaces. The first part of the table deals with general data measured on the bare Si(100)-2x1 surface and on the fully hydrogenated Si(100):H surface extracted from refs. 15, 21 and 22. The DOS peaks voltages measured experimentally for this work on the Si-DBs and in between the Si-DBs are recalled. The second part of the table brings more details about the energies of the PDOS peaks computed on the Si-DB lines as well as with the As atom and allows a comparison with the fully hydrogenated surface. Some values are colored to help the comparison between experimental and theoretical information.

Supplementary References:

[1] Nakatsuji, K.; Takagi, Y.; Komori, F.; Kusuhara, H.; Ishii, A., Electronic states of the clean Ge(111) surface near Fermi energy. Phys. Rev. B **72**, 241308(R) (2005).

[2] Sagisaka, K., Fujita, D. Standing waves on Si(100) and Ge(100) surfaces observed by scanning tunneling microscopy. Phys. Rev. B **72**, 235327 (2005).

[3] Low, T. ; Li, M.F., Yeo, Y.C.; Fan, W.J., Ng, T.; Kwong, D.L. Valence band structure of ultrathin silicon and germanium channels in metal-oxide-semiconductor filed-effect transistors. J. Appl. Phys. **98**, 024504 (2005).

[4] Johansson, L. S. O., Uhrberg, R. I. G., Mårtensson, P., Hansson, G. V. Surface-state band structure of the Si(100)2×1 surface studied with polarization-dependent angle-resolved photoemission on single-domain surfaces, Phys. Rev. B **42**, 1305-1315 (1990).
